# Supplementary material for: Pyruvate Dehydrogenase Kinase 1 inhibition mediated oxidative phosphorylation enhancement in cartilage promotes osteoarthritis progression
Source: BMC Musculoskelet Disord. 2023 Jul 20;24:597. doi: 10.1186/s12891-023-06585-6 (PMC10357736; doi:10.1186/s12891-023-06585-6)
Supplement: Supplementary file 1 — Additional file 1. [file 12891_2023_6585_MOESM1_ESM.zip › BP.Gsea.1653623667859/GOBP_HEART_VALVE_MORPHOGENESIS.html]

Details for gene set GOBP\_HEART\_VALVE\_MORPHOGENESIS[GSEA]

|  || Dataset | 55457\_collapsed\_to\_symbols.cls.cls #OA\_versus\_normal.cls.cls #OA\_versus\_normal\_repos |
| Phenotype | cls.cls#OA\_versus\_normal\_repos |
| Upregulated in class | normal |
| GeneSet | GOBP\_HEART\_VALVE\_MORPHOGENESIS |
| Enrichment Score (ES) | -0.44683513 |
| Normalized Enrichment Score (NES) | -1.7223711 |
| Nominal p-value | 0.0032626428 |
| FDR q-value | 0.12269377 |
| FWER p-Value | 1.0 |
Table: GSEA Results Summary

  

Fig 1: Enrichment plot: GOBP\_HEART\_VALVE\_MORPHOGENESIS      
 Profile of the Running ES Score & Positions of GeneSet Members on the Rank Ordered List

  

| SYMBOL | TITLE | RANK IN GENE LIST | RANK METRIC SCORE | RUNNING ES | CORE ENRICHMENT || 1 | SOX9 | SRY-box transcription factor 9 [Source:HGNC Symbol;Acc:HGNC:11204] | 2089 | 0.351 | -0.1394 | No |
| 2 | TGFB1 | transforming growth factor beta 1 [Source:HGNC Symbol;Acc:HGNC:11766] | 2490 | 0.303 | -0.1506 | No |
| 3 | BMPR1A | bone morphogenetic protein receptor type 1A [Source:HGNC Symbol;Acc:HGNC:1076] | 2745 | 0.275 | -0.1522 | No |
| 4 | GATA4 | GATA binding protein 4 [Source:HGNC Symbol;Acc:HGNC:4173] | 3009 | 0.249 | -0.1563 | No |
| 5 | NFATC1 | nuclear factor of activated T cells 1 [Source:HGNC Symbol;Acc:HGNC:7775] | 3027 | 0.247 | -0.1413 | No |
| 6 | SMAD6 | SMAD family member 6 [Source:HGNC Symbol;Acc:HGNC:6772] | 3460 | 0.206 | -0.1614 | No |
| 7 | ROBO1 | roundabout guidance receptor 1 [Source:HGNC Symbol;Acc:HGNC:10249] | 3737 | 0.182 | -0.1708 | No |
| 8 | ELN | elastin [Source:HGNC Symbol;Acc:HGNC:3327] | 3779 | 0.179 | -0.1623 | No |
| 9 | ACVR1 | activin A receptor type 1 [Source:HGNC Symbol;Acc:HGNC:171] | 3898 | 0.165 | -0.1606 | No |
| 10 | HEY2 | hes related family bHLH transcription factor with YRPW motif 2 [Source:HGNC Symbol;Acc:HGNC:4881] | 4058 | 0.150 | -0.1631 | No |
| 11 | MEF2C | myocyte enhancer factor 2C [Source:HGNC Symbol;Acc:HGNC:6996] | 4218 | 0.135 | -0.1665 | No |
| 12 | TBX5 | T-box transcription factor 5 [Source:HGNC Symbol;Acc:HGNC:11604] | 4748 | 0.091 | -0.2017 | No |
| 13 | HEYL | hes related family bHLH transcription factor with YRPW motif like [Source:HGNC Symbol;Acc:HGNC:4882] | 4820 | 0.086 | -0.2015 | No |
| 14 | SLIT3 | slit guidance ligand 3 [Source:HGNC Symbol;Acc:HGNC:11087] | 5134 | 0.061 | -0.2219 | No |
| 15 | RB1 | RB transcriptional corepressor 1 [Source:HGNC Symbol;Acc:HGNC:9884] | 6013 | -0.006 | -0.2898 | No |
| 16 | OLFM1 | olfactomedin 1 [Source:HGNC Symbol;Acc:HGNC:17187] | 6019 | -0.006 | -0.2898 | No |
| 17 | TGFB2 | transforming growth factor beta 2 [Source:HGNC Symbol;Acc:HGNC:11768] | 6985 | -0.079 | -0.3597 | No |
| 18 | STRA6 | signaling receptor and transporter of retinol STRA6 [Source:HGNC Symbol;Acc:HGNC:30650] | 7107 | -0.089 | -0.3633 | No |
| 19 | MDM2 | MDM2 proto-oncogene [Source:HGNC Symbol;Acc:HGNC:6973] | 7273 | -0.101 | -0.3695 | No |
| 20 | DCHS1 | dachsous cadherin-related 1 [Source:HGNC Symbol;Acc:HGNC:13681] | 7464 | -0.115 | -0.3767 | No |
| 21 | BMP4 | bone morphogenetic protein 4 [Source:HGNC Symbol;Acc:HGNC:1071] | 7816 | -0.142 | -0.3946 | No |
| 22 | HEY1 | hes related family bHLH transcription factor with YRPW motif 1 [Source:HGNC Symbol;Acc:HGNC:4880] | 8213 | -0.172 | -0.4141 | No |
| 23 | TGFBR2 | transforming growth factor beta receptor 2 [Source:HGNC Symbol;Acc:HGNC:11773] | 8241 | -0.174 | -0.4048 | No |
| 24 | SLIT2 | slit guidance ligand 2 [Source:HGNC Symbol;Acc:HGNC:11086] | 8449 | -0.190 | -0.4084 | No |
| 25 | EMILIN1 | elastin microfibril interfacer 1 [Source:HGNC Symbol;Acc:HGNC:19880] | 8826 | -0.220 | -0.4231 | No |
| 26 | SMAD4 | SMAD family member 4 [Source:HGNC Symbol;Acc:HGNC:6770] | 9035 | -0.235 | -0.4239 | No |
| 27 | SOX4 | SRY-box transcription factor 4 [Source:HGNC Symbol;Acc:HGNC:11200] | 9291 | -0.255 | -0.4269 | No |
| 28 | TIE1 | tyrosine kinase with immunoglobulin like and EGF like domains 1 [Source:HGNC Symbol;Acc:HGNC:11809] | 9548 | -0.278 | -0.4285 | Yes |
| 29 | EFNA1 | ephrin A1 [Source:HGNC Symbol;Acc:HGNC:3221] | 9687 | -0.290 | -0.4202 | Yes |
| 30 | SNAI2 | snail family transcriptional repressor 2 [Source:HGNC Symbol;Acc:HGNC:11094] | 9697 | -0.291 | -0.4017 | Yes |
| 31 | NKX2-5 | NK2 homeobox 5 [Source:HGNC Symbol;Acc:HGNC:2488] | 9875 | -0.309 | -0.3951 | Yes |
| 32 | NOS3 | nitric oxide synthase 3 [Source:HGNC Symbol;Acc:HGNC:7876] | 9980 | -0.319 | -0.3822 | Yes |
| 33 | ROCK2 | Rho associated coiled-coil containing protein kinase 2 [Source:HGNC Symbol;Acc:HGNC:10252] | 10648 | -0.388 | -0.4086 | Yes |
| 34 | NOTCH2 | notch receptor 2 [Source:HGNC Symbol;Acc:HGNC:7882] | 10720 | -0.395 | -0.3881 | Yes |
| 35 | BMP2 | bone morphogenetic protein 2 [Source:HGNC Symbol;Acc:HGNC:1069] | 10919 | -0.420 | -0.3758 | Yes |
| 36 | JAG1 | jagged canonical Notch ligand 1 [Source:HGNC Symbol;Acc:HGNC:6188] | 10977 | -0.427 | -0.3521 | Yes |
| 37 | MDM4 | MDM4 regulator of p53 [Source:HGNC Symbol;Acc:HGNC:6974] | 11047 | -0.437 | -0.3287 | Yes |
| 38 | BMPR2 | bone morphogenetic protein receptor type 2 [Source:HGNC Symbol;Acc:HGNC:1078] | 11174 | -0.454 | -0.3086 | Yes |
| 39 | NPPA | natriuretic peptide A [Source:HGNC Symbol;Acc:HGNC:7939] | 11653 | -0.525 | -0.3112 | Yes |
| 40 | TWIST1 | twist family bHLH transcription factor 1 [Source:HGNC Symbol;Acc:HGNC:12428] | 11696 | -0.531 | -0.2795 | Yes |
| 41 | ROCK1 | Rho associated coiled-coil containing protein kinase 1 [Source:HGNC Symbol;Acc:HGNC:10251] | 12046 | -0.605 | -0.2668 | Yes |
| 42 | MTOR | mechanistic target of rapamycin kinase [Source:HGNC Symbol;Acc:HGNC:3942] | 12047 | -0.605 | -0.2270 | Yes |
| 43 | GJA5 | gap junction protein alpha 5 [Source:HGNC Symbol;Acc:HGNC:4279] | 12095 | -0.619 | -0.1899 | Yes |
| 44 | GATA3 | GATA binding protein 3 [Source:HGNC Symbol;Acc:HGNC:4172] | 12276 | -0.670 | -0.1598 | Yes |
| 45 | SNAI1 | snail family transcriptional repressor 1 [Source:HGNC Symbol;Acc:HGNC:11128] | 12365 | -0.696 | -0.1208 | Yes |
| 46 | ADAMTS9 | ADAM metallopeptidase with thrombospondin type 1 motif 9 [Source:HGNC Symbol;Acc:HGNC:13202] | 12428 | -0.717 | -0.0784 | Yes |
| 47 | NOTCH1 | notch receptor 1 [Source:HGNC Symbol;Acc:HGNC:7881] | 12555 | -0.776 | -0.0371 | Yes |
| 48 | CCN1 | cellular communication network factor 1 [Source:HGNC Symbol;Acc:HGNC:2654] | 12802 | -0.970 | 0.0076 | Yes |
Table: GSEA details [plain text format]

  

Fig 2: GOBP\_HEART\_VALVE\_MORPHOGENESIS      
 Blue-Pink O' Gram in the Space of the Analyzed GeneSet

  

Fig 3: GOBP\_HEART\_VALVE\_MORPHOGENESIS: Random ES distribution      
 Gene set null distribution of ES for **GOBP\_HEART\_VALVE\_MORPHOGENESIS**
